# 10-year-old girl with life-threatening idiopathic systemic capillary leak syndrome: a case report

**DOI:** 10.1186/1471-2431-14-137

**Published:** 2014-05-31

**Authors:** Tadashi Iwasa, Hiroyuki Ohashi, Kentaro Kihira, Yuhki Koike, Kohei Otake, Mikihiro Inoue, Hirofumi Sawada, Hidemi Toyoda, Yoshihiro Komada

**Affiliations:** 1 Department of Pediatrics, Mie University Graduate School of Medicine, 2-174 Edobashi, Tsu City, Mie Prefecture 514-8507, Japan; 2 Department of Gastrointestinal and Pediatric Surgery, Mie University Graduate School of Medicine, 2-174 Edobashi, Tsu City, Mie Prefecture 514-8507, Japan

**Keywords:** Idiopathic systemic capillary leak syndrome, Vascular endothelial damage, Methylprednisolone pulse, Theophylline, Terbutaline

## Abstract

**Background:**

Idiopathic systemic capillary leak syndrome (ISCLS) is a rare disorder, characterized by episodic life-threatening hypotension, hypoalbuminemia, and hemoconcentration.

**Case presentation:**

A 10-year-old girl presented with abdominal pain, vomiting, diarrhea, fever and developed generalized edema a day after admission. Clinical and laboratory findings were consistent with ISCLS. She received aggressive fluid replacement, methylprednisolone pulse (30 mg/kg/day), high-dose intravenous immunoglobulin (IVIG, 2 g/kg/day) and plasma exchange in acute phase. She received fasciotomy of bilateral lower extremities as she developed complications of compartment syndrome. Since there were two episodes of ISCLS attacks, theophylline and terbutaline were initiated for prevention of attacks and then the remission is currently maintained. Because of high fatality rate in ISCLS, prompt diagnosis and intervention are very important.

**Conclusion:**

We describe here, a rare case of pediatric ISCLS. ISCLS should be considered as a differential diagnosis, when the patient presents with unexplained or sudden hypovolemic shock. Reports on pediatrics ISCLS are very few, and accumulation of similar case reports is needed.

## Background

Idiopathic Systemic Capillary Leak Syndrome (ISCLS), also known as Clarkson’s disease, is a very rare disorder, characterized by recurrent episodes of severe hypotension, hypoalbuminemia and hemoconcentration [1]. Attacks of ISCLS demonstrate three phases: (1) prodrome, (2) hypovolemia with weight gain and (3) hypervolemia with fluid overload and polyuria often complicated by pulmonary edema. Compartment syndrome, leading to rhabdomyolysis is a serious complication of ISCLS [2].

## Case presentation

A 10-year-old girl; previously healthy, and with an unremarkable clinical history, presented with abdominal pain, diarrhea and the axillary temperature was 37.6°C. She had no pruritus. She consulted a clinic with hypotension, tachycardia (156 beats/minute) and somnolence tendency. Rapid fluid infusion was administered immediately, and blood pressure was recovered to 120/68 mmHg in fifteen minutes. She was later transferred to a local hospital where she received rapid infusion (0.3 L per hour) as systolic blood pressure was 40 mmHg. Since hypovolemic shock was suspected, she was transferred to our hospital. She had somnolence tendency and pallor, with blood pressure 100/60 mmHg, tachycardia (180 beats/minute), fever (38.1°C) and body weight 34.3 kg (34.0 kg before admission). Laboratory data indicated hemoconcentration, hypoalbuminemia, renal dysfunction, leukocytosis and elevated C-reactive protein (Table 1). Arterial blood gas analysis showed metabolic acidosis. Urinalysis on admission revealed proteinuria. It was transient because the following urinalysis revealed no proteinuria. Chest X-ray demonstrated cardio-thoracic ratio 45% with no pleural effusion. Antibiotics were administered on suspicion of bacterial infection. Echocardiography revealed modest pericardial effusion with ejection fraction of 50%. Computed tomography (CT) showed massive ascites and small intestinal dilatation. Because systolic blood pressure had decreased to 50 mmHg, she received massive infusion (saline solution), albumin, fresh frozen plasma and vasopressor (dopamine). On day 1 systolic blood pressure could be maintained at 70 to 90 mmHg. CT was performed again and demonstrated the pleural effusion and abdominal distension with massive ascites. She was intubated and received mechanical ventilation. On day 2 she developed severe hypoalbuminemia (1.8 g/dl) and weight gain (40.7 kg). Severe ISCLS was suspected. She received methylprednisolone (30 mg/kg/day), high-dose IVIG (2 g/kg/day) and plasma exchange. Because she presented with the oliguria (300 mL/day) and hyperkalemia (6.6 mEq/L) by acute renal failure, continuous hemodiafiltration (CHDF) was started. She had complications of compartment syndrome of lower extremities, which led to rhabdomyolysis. Therefore she underwent fasciotomy. On day 3 hemodynamic state was stable and serum albumin level was maintained at or above 2.5 mg/dL. Interleukin (IL) -6 and granulocyte colony-stimulating factor (G-CSF) were elevated in plasma (Shown in Table 1). Vascular endothelial growth factor (VEGF) was normal in plasma, but was elevated in ascites. Since she had second attack of ISCLS on day 26, combined prophylactic therapy with terbutaline and theophylline was initiated for prevention. She had anuria (20–80 ml/day) for 55 days and subsequently urine output was gradually increased. She was discharged with having mild sensory and motor disturbance of lower extremities. Currently, she is healthy without episodes of ISCLS attack during follow-up for over 1 year.

**Table 1:** Laboratory data on admission

WBC	41 × 10^3^/μL	(3.9-6.60 × 10^3^/μL)	C1-INH	150%	(70–130%)
RBC	7.99 × 10^6^/μL	(4.08-4.81 × 10^6^/μL)	C3	23.6 IU/L	(65.0-141.0 IU/L)
Hb	23.3 g/dl	(12.0-14.7 g/dl)	C4	6.1 IU/L	(13.0-40.0 IU/L)
Ht	64.4%	(37.0-44.6%)	CH50	15.1 IU/L	(31–48 IU/L)
PLT	25.7 × 10^4^/μL	(18.0-36.5 × 10^4^/μL)	ANA	<40	(<40)
			VEGF	<20 pg/ml	(<20 pg/ml)
			IL-6	85.8 pg/ml	(<420 pg/ml)
Alb	2.3 g/dL	(3.5-5.0 g/dL)	G-CSF	217 pg/ml	(<39 pg/ml)
BUN	42 mg/dL	(8–20 mg/dL)			
CRE	2.1 mg/dL	(0.42-0.68 mg/dL)	Urinalysis		
Na	130 mEq/L	(135–145 mEq/L)	protein	100 mg/dL	
K	4.8 mEq/L	(3.3-5.0 mEq/L)	blood	3+	
Cl	98 mEq/L	(96–109 mEq/L)	RBC	>100 /HPF	
Ca	7.6 mg/dL	(8.5-10.5 mg/dL)	Bence-Jones Protein	(−)	
CPK	399 U/L	(12–170 U/L)			
CRP	3.24 mg/dL	(0–0.30 mg/dL)	Culture (Blood, Urine, Stool): (−)		

C1-INH: C1-esterase inhibitor, ANA: antinuclear antibody, VEGF: vascular endothelial growth factor IL-6, G-CSF: granulocyte colony-stimulating factor.

## Conclusions

Only 10 cases of ISCLS in children have been reported to date [3-11]. These attacks occurred at ages ranging from 5 months to 8 years. Most of the cases were severe or fatal. All patients in reports were previously healthy and only one had a family history of ISCLS. ISCLS should be included in the differential diagnosis of sudden hypovolemic shock with general edema (anaphylaxis, C1-esterase inhibitor deficiency, nephrotic syndrome). Pathogenesis and pathophysiology of ISCLS are relatively unknown. Reports suggest that VEGF and angiopoietin 2 (Ang2) contribute to endothelial contraction and might be associated with pathogenesis [12]. Elevated levels of cytokines and chemical mediators (G-CSF, IL-6, IL-8 and monocyte chemotactic protein-1: MCP-1) were reported in some cases [13]. G-CSF and VGEF levels might be a used as biomarkers for the severity and for monitoring the clinical course in ISCLS [14,15]. In the present case, G-CSF was elevated, but VEGF was normal in plasma. Surprisingly, VEGF in ascites was elevated. There was a case report that did not show increase of VEGF in plasma as the present case, but the reason is unknown [16]. Further investigations of various biomarkers, which might play an important role, are needed for clarify the pathophysiology of ISCLS. Although there are no established therapies in acute phase, corticosteroids and IVIG has been used to treat ISCLS attacks [8,17]. Recently, it has been reported that a patient with ISCLS after stem cell transplantation was received anti-VEGF antibody bevacizumab despite low serum VEGF level, and improved within 48 hours [16]. In pediatric case, the tumor necrosis factor alpha (TNF-α) antagonist infliximab was administered when serum TNF-α was increased, and clinical course was developed dramatically [6]. In present case, methylprednisolone and IVIG could not improve symptoms and the effect of these treatments was limited. Therefore, appropriate fluid management in acute phase is most important. Prophylactic treatment with theophylline and terbutaline as well as IVIG has been shown to reduce frequency and severity of ISCLS attacks, which could be effective in the present case [8,18].

In conclusion, ISCLS should be considered in the differential diagnosis when unexplained hypovolemic shock is observed. The diagnostic evaluation should be performed concurrently with initial fluid management. Immediate intervention should be started because ISCLS has a high fatality rate.

### Consent

Written informed consent was obtained from the patient’s parents for publication of this Case report and any accompanying images. A copy of the written consents is available for review by the Editor of this journal.

## Abbreviations

ISCLS: Idiopathic systemic capillary leak syndrome; IVIG: Intravenous immunoglobulin; IL: Interleukin; G-CSF: Granulocyte colony-stimulating factor; VEGF: Vascular endothelial growth factor; CHDF: Continuous hemodiafiltration; C1-INH: C1-esterase inhibitor; MCP-1: Monocyte chemotactic protein-1; TNF-α: Tumor necrosis factor alpha.

## Competing interests

The authors declare that they have no competing interests.

## Authors’ contributions

All authors contributed greatly to write this article. TI, HO, KK, YK, KO, MI and HS were involved in the diagnostic and clinical management of this patient. All authors read and approved the final manuscript.

## Pre-publication history

The pre-publication history for this paper can be accessed here:

http://www.biomedcentral.com/1471-2431/14/137/prepub
